# RNA Polymerase II Binding Patterns Reveal Genomic Regions Involved in MicroRNA Gene Regulation

**DOI:** 10.1371/journal.pone.0013798

**Published:** 2010-11-02

**Authors:** Guohua Wang, Yadong Wang, Changyu Shen, Yi-wen Huang, Kun Huang, Tim H. M. Huang, Kenneth P. Nephew, Lang Li, Yunlong Liu

**Affiliations:** 1 Center for Computational Biology and Bioinformatics, Indiana University School of Medicine, Indianapolis, Indiana, United States of America; 2 School of Computer Science and Technology, Harbin Institute of Technology, Harbin, China; 3 Division of Biostatistics, Indiana University School of Medicine, Indianapolis, Indiana, United States of America; 4 Division of Human Cancer Genetics, Ohio State University, Columbus, Ohio, United States of America; 5 Department of Molecular Virology, Immunology, and Medical Genetics, Ohio State University, Columbus, Ohio, United States of America; 6 Comprehensive Cancer Center, Ohio State University, Columbus, Ohio, United States of America; 7 Department of Biomedical Informatics, Ohio State University, Columbus, Ohio, United States of America; 8 Medical Sciences, Indiana University School of Medicine, Bloomington, Indiana, United States of America; 9 Departments of Cellular and Integrative Physiology, Indiana University School of Medicine, Indianapolis, Indiana, United States of America; 10 Indiana University Simon Cancer Center, Indiana University School of Medicine, Indianapolis, Indiana, United States of America; 11 Department of Medical and Molecular Genetics, Indiana University School of Medicine, Indianapolis, Indiana, United States of America; 12 Center for Medical Genomics, Indiana University School of Medicine, Indianapolis, Indiana, United States of America; University of Calgary, Canada

## Abstract

MicroRNAs are small non-coding RNAs involved in post-transcriptional regulation of gene expression. Due to the poor annotation of primary microRNA (pri-microRNA) transcripts, the precise location of promoter regions driving expression of many microRNA genes is enigmatic. This deficiency hinders our understanding of microRNA-mediated regulatory networks. In this study, we develop a computational approach to identify the promoter region and transcription start site (TSS) of pri-microRNAs actively transcribed using genome-wide RNA Polymerase II (RPol II) binding patterns derived from ChIP-seq data. Based upon the assumption that the distribution of RPol II binding patterns around the TSS of microRNA and protein coding genes are similar, we designed a statistical model to mimic RPol II binding patterns around the TSS of highly expressed, well-annotated promoter regions of protein coding genes. We used this model to systematically scan the regions upstream of all intergenic microRNAs for RPol II binding patterns similar to those of TSS from protein coding genes. We validated our findings by examining the conservation, CpG content, and activating histone marks in the identified promoter regions. We applied our model to assess changes in microRNA transcription in steroid hormone-treated breast cancer cells. The results demonstrate many microRNA genes have lost hormone-dependent regulation in tamoxifen-resistant breast cancer cells. MicroRNA promoter identification based upon RPol II binding patterns provides important temporal and spatial measurements regarding the initiation of transcription, and therefore allows comparison of transcription activities between different conditions, such as normal and disease states.

## Introduction

MicroRNAs are small (∼22 nucleotides) non-coding RNAs known to regulate the expression of target genes by promoting mRNA degradation and suppressing translation [1], [2], [3], [4]. The discovery of microRNAs has identified new mechanisms of gene regulation that play critical roles in multiple biological processes, including cell cycle control, cell growth and differentiation, apoptosis, embryo development, and so on [5], [6], [7], [8], [9]. While several hundred precursor microRNAs (pre-miRNAs) and mature microRNAs have been sequenced and annotated in human, mouse, rat, and drosophila genomes [10], most primary microRNAs (pri-miRNAs), which are transcribed by RNA Polymerase II (RPol II) and further processed to pre-miRNAs in the nucleus, have yet to be identified.

The regulation of microRNA biogenesis consists of three major steps 1) pri-miRNA transcribed by RNA polymerase II and III; 2) microRNA maturation, including nuclear cleavage of the pri-miRNA to precursor microRNA and nucleocytoplasmic export, and 3) RISC (RNA-induced silencing complex) assembly that converts pre-miRNAs to mature microRNAs [11], [12], [13], [14]. Although microRNA biogenesis can be regulated at any of these three steps, identifying microRNA transcription start sites and regulatory regions is critical to understanding transcription factor-mediated regulation. Toward this objective, previous studies have used individual genome features, such as transcription factor binding site prediction [15], sequence conservation among multiple species [16], expressed sequence tags (ESTs) [17], and genome wide binding patterns of RPol II [18]. More recently, epigenetic marks, including trimethylation of lysine 4 at histone H3 (H3K4me3), have been shown to be highly localized at gene promoters [19], including microRNA promoter regions [20]. However, many of the previously identified pri-miRNAs have yet to be fully or accurately annotated, and transcriptional mechanisms governing microRNA regulation remain incompletely understood.

In the current study, we designed a computational approach using genome-wide RPol II binding patterns to identify the promoter region and transcription start site of pri-miRNAs that are actively transcribed. Because transcriptional regulation of most intronic microRNAs is controlled by promoter sequences of the corresponding host protein-coding genes [21], we focused on “intergenic” microRNA, i.e., microRNAs residing outside of intronic regions of a host gene and previously demonstrated to be primarily transcribed by RPol II [14]. Our model can be used to scan the upstream regions of annotated microRNAs and identify putative transcription start sites and active promoters, providing a statistical framework for evaluating sensitivity and specificity of the model prediction and for self-correcting experimental variation in RPol II binding signals, thus making it possible to compare microRNA promoter signals under different biological conditions.

## Results

The goal of this study was to use ChIP-seq derived RPol II binding data to identify promoter regions of microRNAs actively transcrbied. We develop a computational model to assess changes in microRNA transcription and genome-wide RPol II binding patterns in steroid hormone-treated breast cancer cells. Four biological conditions and two breast cancer cell lines were utilized: vehicle-treated (control) hormone-dependent MCF7, the anti-estrogen resistant MCF7 subline MCF7-T (tamoxifen resistant, described previously in Fan et al., 2006 [22]) and MCF7 and MCF7-T treated with 17-β-estradiol (E2) for three hours. RPol II patterns were determined using ChIP-seq (chromatin-immunoprecipitation followed by next generation sequencing Illumina 1G platform). ChIP-seq fragments that had either a poor quality score or could not be mapped to a unique genomic locus were removed; this analysis resulted in 5-7 million DNA fragments for each of the four conditions (MCF7+/−E2; MCF7-T+/−E2). In addition, mRNA expression levels were determined for the same conditions using Affymetrix Human Genome U133 plus 2 GeneChip [22].

**Figure 1:**
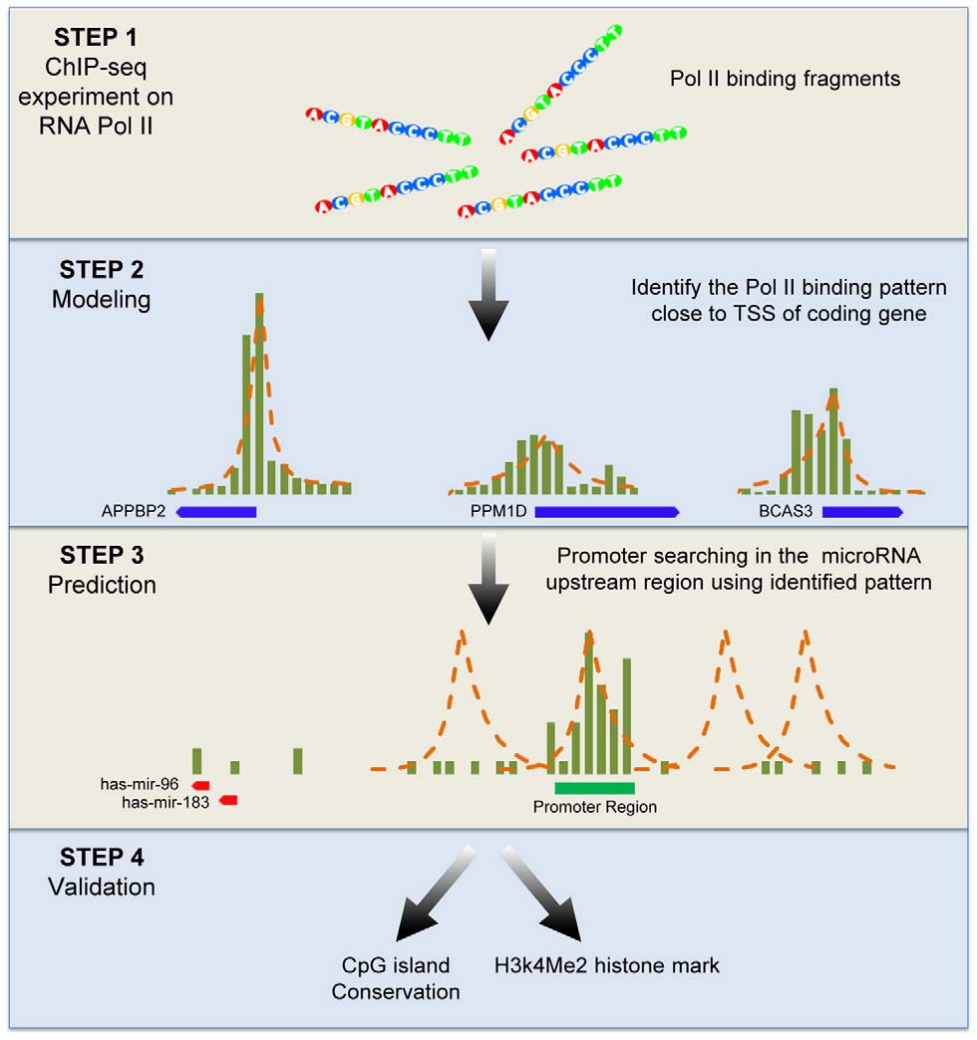
Procedure for identifying microRNA promoters. The overall procedure includes four major steps: (1) using ChIP-seq experiment to identify genome-wide RPol II binding patterns; (2) characterizing the features of the RPol II binding pattern surrounding the transcription start site (TSS) of coding genes; and (3) scaning genomic regions upstream of all annotated microRNAs containing similar binding patterns as protein coding genes; and (4) validating the identified microRNA regulatory regions.

The overall procedure to systematically identify regulatory regions of human microRNA genes is demonstrated in Figure 1. As our approach assumes that the distribution of RPol II binding patterns around the transcription start site (TSS) of microRNA and protein coding genes are similar, we first designed a statistical model to mimic RPol II binding patterns around the TSS of well-annotated promoter regions of highly expressed protein coding genes. To identify promoter regions of expressed microRNAs, we systematically scanned the upstream regions of all the intergenic microRNAs searching for genomic regions statistically similar to RPol II binding patterns around the TSS of the coding genes. We then validated our findings by examining the conservation, GC content, and activating histone marks in the identified promoter regions.

### RPol II binding patterns around the TSS of expresed protein coding genes

**Figure 2:**
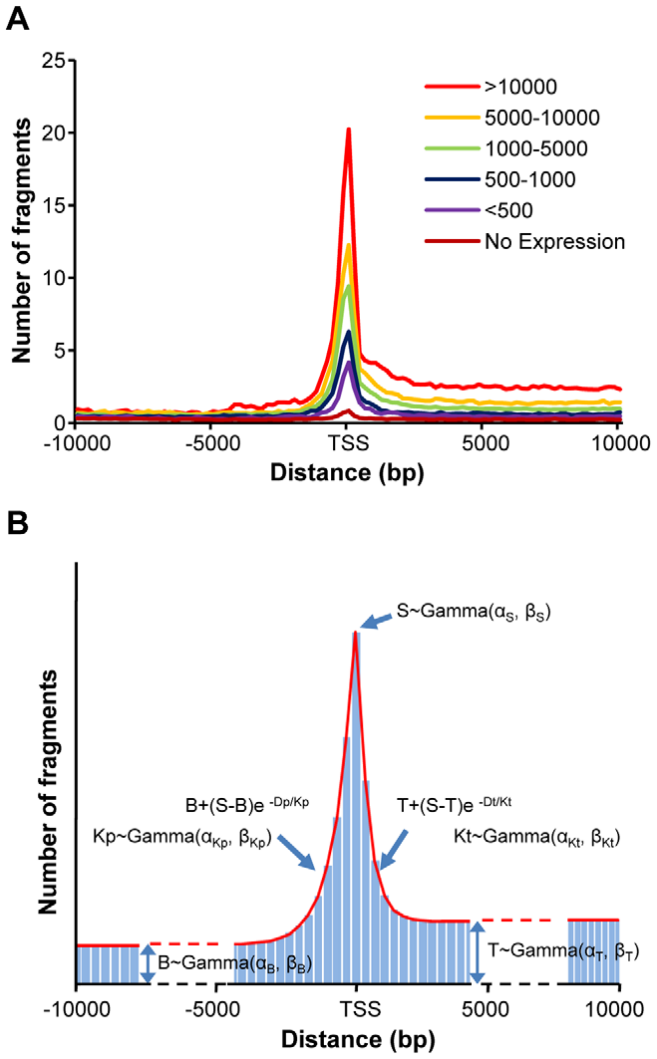
RPol II binding fragments surrounding TSS of protein coding genes. (A) The ChIP-Seq-derived RPol II binding pattern around theTSS of protein coding gene in MCF7 cells. Protein-coding genes (n = 16,000) were separated into six groups, based upon their expression levels, which are measured using microarray experiments. (B) A statistical model of RPol II binding pattern surrounding the TSS of expressed genes. The adjacent genomic regions are divided into multiple 200-bp bins, in which the number of RPol II fragments is assumed to follow *Poisson* distribution. For each gene, the overall binding pattern can be characterized by five hidden variables, including three variables describing the expected number of fragments in the background region (B), the transcript region (T), and the bin that contains TSS (S), and two variables modeling the signal decay rates in both upstream and downstream of the TSS (K_p_ and K_t_). Each hidden variable follows a Gamma distribution genome-wide.

We first examined the RPol II binding pattern around the TSS of expressed protein coding genes, whose express levels are evaluated using Affymetrix Human Genome U133 plus 2 GeneChip [22]. The signal intensities were extracted using Affymetrix Microarray Suite 5.0 (MAS5). MAS5 uses a non-parametric statistical test (Wilcoxon signed rank test) to produce a detection call (Absent (A), Present (P) or Marginal (M)) for each probe set, based on whether the hybridization signal of perfect-matched probes is significantly greater than their corresponding mismatches. For the genes whose expression levels can be reliably detected (Present), we calculated the total number of RPol II-derived fragments within 5,000 base pairs (bp) upstream and downstream of the TSS, producing a RPol II binding landscape in the regulatory regions of expressed genes. Not surprisingly, we observed significant enrichment of the RPol II signal on top of the TSS (Figure 2A), which gradually declines towards both upstream and downstream (transcript) regions. In the transcript region (downstream), higher steady state RPol II signals are maintained compared to upstream regions, eventually entering intergenic regions (background). We further sub-classified expressed genes based upon their expression levels, and genes with higher expression levels tended to display higher the average RPol II signals around the TSS (Figure 2A). For the coding genes with undetectable (Absent) expression levels, RPol II enrichment around the TSS was markedly lower; the minor enrichment of RPol II signal around TSS is perhaps due to quiescent mechanisms such as RPol II staling.

To mimic RPol II binding patterns surrounding the TSS of expressed genes, a graphic model was used (Figure 2B). Intuitively, for any given genomic region, the total number of RPol II binding fragments should follow a *Poisson* distribution, and we therefore focused on 200-bp genomic intervals. For each protein coding gene, the genomic region was classified into three categories: one central interval (centered at the TSS), 25 upstream intervals, and 25 downstream intervals. The *Poisson* parameter *λ* for each interval was based on the transcription level of the gene being studied and the location of the interval relative to the transcription start site. As shown in Figure 2B, five factors were used to model the *Poisson* parameter *λ*: S – the number of RPol II binding fragments in the central interval (location of the TSS); T – the number of RPol II binding fragments in the steady transcript region; B – the number of RPol II binding fragments in the steady background region and; K_p_ and K_t_ – decay factors of the number of RPol II binding fragments in the promoter and transcription regions, respectively. These five factors each follow a *Gamma* distribution genome wide among all the expressed genes; therefore, we assume that the RPol II binding patterns around the TSS of expressed genes were determined by 10 *Gamma* parameters **Φ** (see methods).

For each of the four biological samples (MCF7+/−E2; MCF7-T+/−E2), the 10 parameters were identified by maximizing the posterior probability defined as 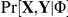 (for details methods see Appendix S1), where **X** denotes the number of detected RPol II-ChIP-seq fragments; **Y** stands for the five hidden variables that determine the Poisson parameter **λ_i_** (Eq. 2) for each gene; and **Φ** represents the ten parameters describing the distribution of the five hidden variables. The optimal estimations for the 10 parameters in four conditions (MCF7+/−E2; MCF7-T+/−E2) are shown in Table S1. In all four samples, the expected promoter decay factors K_p_ were larger than the expected transcription decay factor K_t_, indicating that RPol II binding quantities reached steady state levels more rapidly in the transcript region (downstream of the TSS) than in the promoter (upstream) region. If a higher-than-background RPol II binding implies additional interaction(s) with other transcription factors, the longer regulatory region upstream of the TSS supports the concept that transcription factors initiate transcription by binding regulatory elements upstream of the TSS; this is due to the nature of ChIP-seq experiment, in which both protein-DNA and protein-protein interactions will be cross linked. We also observed that the expected quantities of RPol II in the transcript region (T) were higher than the intergenic region (B), indicating constant transcriptional activity in the expressed genes.

### Predictive power of RPol II binding pattern and transcriptional activity

**Figure 3:**
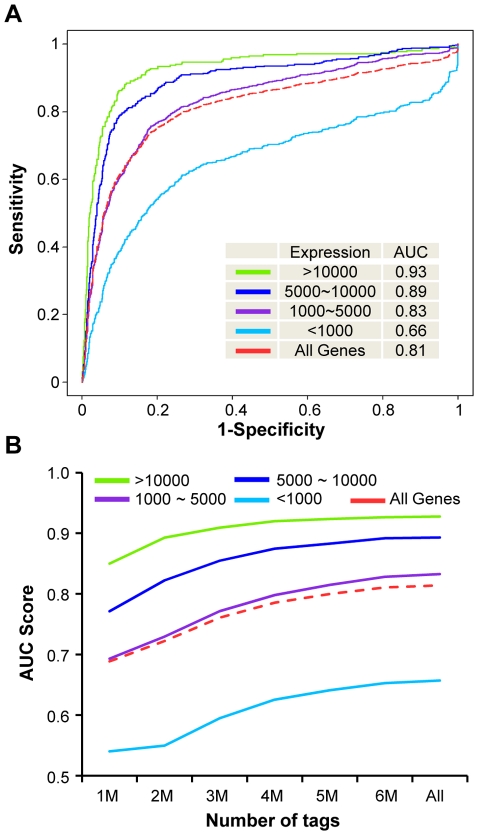
The ROC curve and saturation analysis for TSS prediction of protein coding genes with different expression levels. (A) The ROC curve shows the sensitivity and specificity of the TSS prediction for genes with different expression levels. Genes were separated into four groups, according to expression level. For each group and total genes, the TSS was sorted by score ΔF, and the rate of false predictions (X-axis) and true predictions (Y-axis) was plotted for each possible score prediction threshold. The area under the curve (AUC) for each gene group is shown, computed by extending each plot to the upper right corner. Gene group is shown by a dotted line. (B) The saturation analysis demonstrates the effects of sequencing depth to the prediction. X-axis indicates the number of randomly selected reads from the whole data set, and Y-axis shows the AUC score for identifying actively transcribed promoters for the genes with different levels of expression.

To test the predictive power of our model for identifying microRNA promoter regions, we constructed a “gold standard” by focusing only on genes with lengths of open reading frame greater than 10,000-bp and with no other genes present within 10,000-bp of the TSS. These criteria avoid potential bias due to the transcriptional activities of other genes, which could result in an over estimation of the number of RPol II binding sites; this analysis results in 4007 expressed genes (Present on the Affymetrix array) and 2134 unexpressed genes (Absent genes) in MCF7 cells. To identify model parameters, we randomly selected ¼ of the expressed genes, and the remaining ¾ of expressed genes and all the unexpressed genes were used as positive and negative control sets, respectively. Based upon the parameters **Φ** identified from 1002 genes in the training sets, one score was calculated for each gene by comparing the probability that the RPol II binding pattern around its TSS fits expressed genes rather than genome-wide unexpressed regions (Eq. 3 in the Methods); this is evaluated by the ratio of the likelihood from distributions of expressed genes and background regions, respectively. The probability of fitting the genome-wide unexpressed regions was calculated by assuming that the RPol II binding signals were from intergenic background regions. Our model using RPol II binding patterns around the TSS appeared to accurately distinguish between expressed and unexpressed genes. The area under the curve (AUC) in the Recursive Operating Characteristics (ROC) reached 0.81 in differentiating all the expressed genes in the test set and unexpressed genes (Figure 3A), and the predictive power of this approach increased with gene expression level (Figure 3A), reaching 0.93 for genes signal intensity levels >10,000 and unexpressed genes in the Affymetrix array. The AUC dropped to 0.66 for genes with signal intensities <1,000.

To assess whether the RPol II sequencing depth is adequate in identifying active promoter regions, we performed saturation analysis by analyzing how the prediction power change when only a subset of sequencing reads are used for prediction. The same 4007 expressed genes and 2134 unexpressed genes in MCF7 cells were used for saturation analysis. The AUC score was calculated by randomly selecting 1 million to 6 million reads (Figure 3B). The predictive power of our approach increased with the sequence depth and reached a saturation point with more than 4 million reads. Similar results were achieved for other conditions (Figure S1).

### Identification of microRNA promoters

The objective of this study was to identify the TSS and promoter regions of pri-microRNAs by searching for RPol II binding patterns similar to those seen in expressed protein-coding genes in the upstream regions of annotated mature microRNAs (see methods for details). In brief, for each microRNA, we searched the TSS of the primary microRNA up to 10,000-bp upstream of the mature microRNA. Starting from the 5′-end of the annotated mature microRNA [10], we calculated the number of RPol II-targeted DNA fragments detected in every 200-bp genomic interval. For each interval within 10,000-bp upstream of the mature microRNA, the probability that it contains a TSS was calculated by comparing whether the RPol II binding patterns in the surrounding bins fit the patterns deduced in the expressed coding genes (Eq 3), defined as ΔF. We selected the interval with the largest ΔF score as a potential TSS-containing bin. To evaluate whether the microRNA was actively transcribed, a false discovery rate (FDR) was calculated by comparing this score (ΔF) to the values derived using RPol II binding patterns around unexpressed genes. Here, the promoter regions of unexpressed genes were used as background to estimate the FDR. This background can also be estimated using randomly selected genomic regions. A lower FDR indicated a higher possibility that a particular microRNA was actively transcribed in the respective biological system.

We focused our study on 419 intergenic microRNAs obtained from miRBase microRNA sequence database (version 11.0). The intronic microRNAs, based upon human RefSeq gene annotation (hg18 genome assembly, [23]), were eliminated from the analysis, because they might co-transcribe with host genes. Using an FDR ≤0.2, we identified promoter regions for 49 and 68 microRNAs actively transcribed in vehicle- and E2-treated MCF7cells, respectively (Table S2). In the tamoxifen-resistant cells, 61 and 68 microRNAs were identified in vehicle and E2-treated MCF7-T cells (Table S2). This list contains 72 microRNAs that were detected in at least one sample, 47 of which (65%) were present in all four samples; these 72 microRNAs are from 46 microRNA clusters [10].

**Figure 4:**
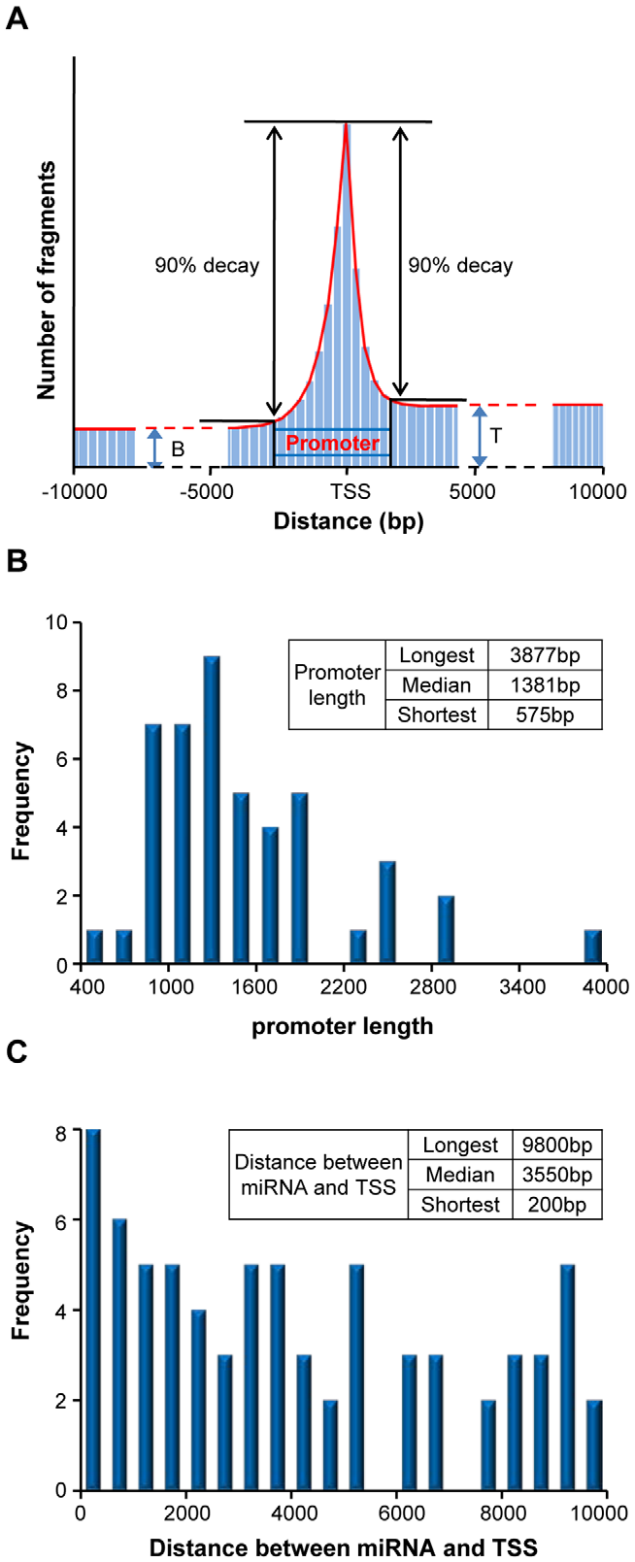
Features of identified microRNA regulatory regions. (A) Schematics of the definition of microRNA promoter region. (B) Histogram illustrating promoter length of the 46 microRNA clusters. (C) Histogram illustrating the distance between 72 mature microRNAs and their predicted microRNA TSS.

Based on the assumption that RPol II binding enrichment around the TSS may be due to the interaction with transcription factors in the regulatory region, for each microRNA, we considered genomic regions with less than a 90% RPol II signal decay compared to the ones in TSS-bin as a potential regulatory region (Figure 4A). For the 46 microRNA clusters detected in at least one sample, the width of the regulatory regions demonstrated significant variation (Figure 4B). The median value of the width of regulatory region was 1381-bp, with longest and shortest widths of 3877-bp and 575-bp, respectively. In addition, we also observed a wide range of genomic distances between the identified TSS and their corresponding microRNA (100's–10000's bp range; Figure 4C), with a median distance of 3550-bp. Such findings are consistent with other studies using sequence features [17] or other types of genomic data [24].

### RPol II binding patterns reveal microRNA predisposition in tamoxifen-resistant breast cancer cells

**Figure 5:**
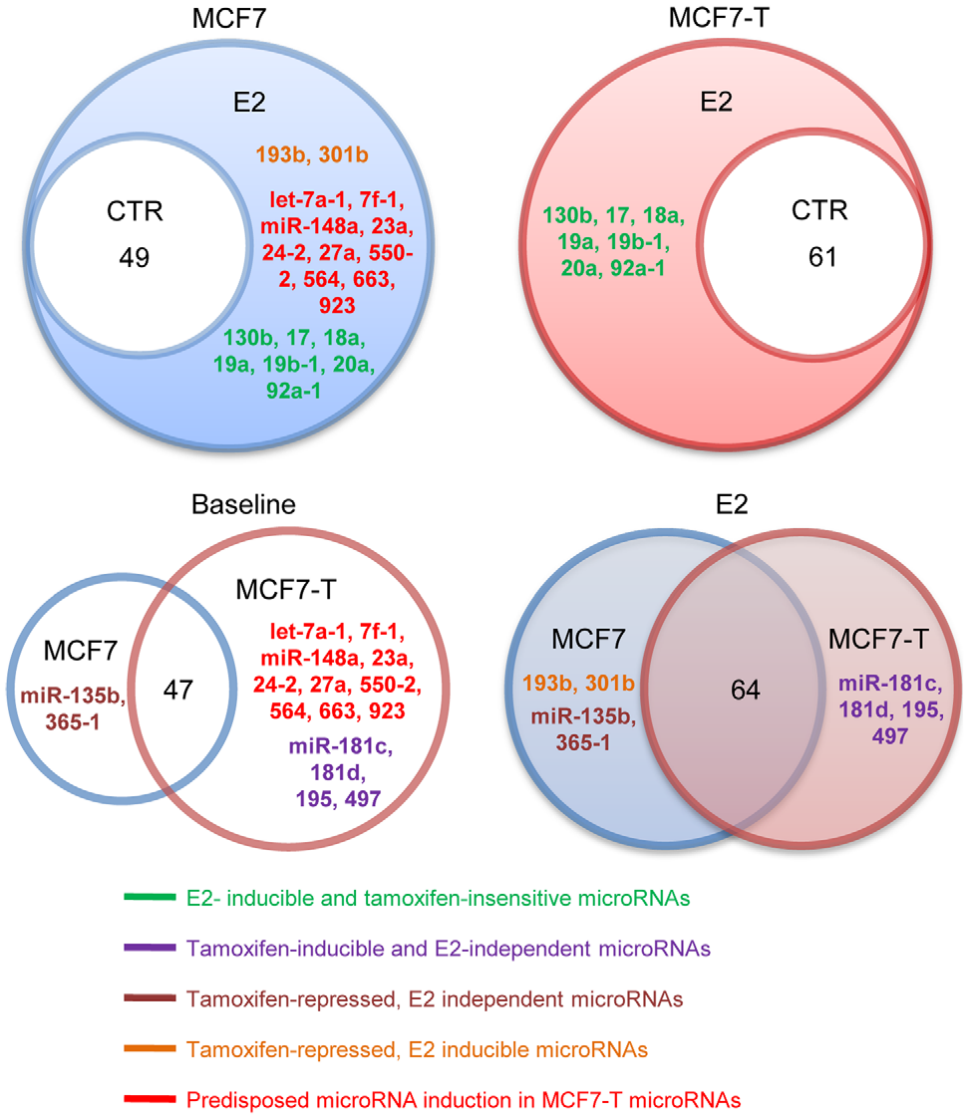
Venn diagram of differentially transcribed microRNAs in breast cancer cells. With FDR ≤0.2, the identified active transcription of microRNAs in four statistical comparisons, MCF7 control vs. MCF7 after treatment with 17β-estradiol (E2 treatment), MCF7-T control vs. MCF7-T after E2 treatment, MCF7 control vs. MCF7-T control, and MCF7 after E2 treatment vs. MCF7-T after E2 treatment. The differentially transcribed microRNAs in each comparison are showed in the middle of the figure.

In MCF7 cells, 49 microRNAs were actively transcribed (FDR ≤0.2), and active transcription of an additional 19 microRNAs was seen after E2 stimulation (Figure 5). Among the 19 E2-induced microRNAs in MCF7 cells, 10 were constitutively active in vehicle-treated MCF7-T cells, and 7 (out of19) were E2- inducible in MCF7-T (Figure 5). These 7 microRNAs were a subset of the E2-induced microRNAs in MCF7 cells, demonstrating that their induction was independent of tamoxifen resistance. These results suggest that the 10 E2-inducible microRNAs in MCF7 cells, which became constitutively upregulated in the MCF7-T cells, may contribute to loss of estrogen sensitivity and acquisition of the antiestrogen resistant pheonotype. Surprisingly, E2 treatment did not repress transcriptional activity of any microRNAs, both in MCF7 and MCF7-T cells. This suggests that decreased expression of previously reported E2-suppressed microRNAs [25] was more likely to be regulated in the RNA processing level (microRNA maturation), rather than on the transcriptional initiation level.

We further classified all 72 microRNAs into the following six categories:

#### MicroRNAs identified in all four samples

This category contains 47 microRNAs that are constitutively transcribed in both MCF7 and MCF7-T cells (no effect of E2 treatment, Table S2);

#### E2- inducible, tamoxifen-insensitive

This category contains 7 microRNAs induced by E2 in both MCF7 and MCF7-T cells (hsa-miR-130b, hsa-miR-17, hsa-miR-18a, hsa-miR-19a, hsa-miR-19b-1, hsa-miR-20a, and hsa-miR-92a-1).

#### Tamoxifen-inducible, E2-independent

This category contains 4 microRNAs (hsa-miR-181c, hsa-miR-181d, hsa-miR-195, and hsa-miR-497) that are transcribed in control and E2-treated MCF7-T but not expressed MCF7 cells.

#### Tamoxifen-repressed, E2 independent

Two microRNAs, hsa-miR-135b and hsa-miR-365-1, were transcribed in both control and E2-treated MCF7 cells but not in either of the MCF7-T groups. Transcription of these two microRNAs is thus suppressed by the tamoxifen treatment and independent of E2 treatment.

#### Tamoxifen-repressed, E2 inducible

Two microRNAs, hsa-miR-193b and hsa-miR-301b, were induced by E2 in MCF7 but not in MCF7-T cells.

#### Predisposed microRNA induction in MCF7-T

Ten microRNAs, hsa-let-7a-1, hsa-let-7f-1, hsa-miR-148a, hsa-miR-23a, hsa-miR-24-2, hsa-miR-27a, hsa-miR-550-2, hsa-miR-564, hsa-miR-663, and hsa-miR-923, were induced by E2 treatment of MCF7 cells, but showed a similar upregulated level of expression in both vehicle- and E2-treated MCF7-T, suggesting that the acquisition of tamoxifen resistance is associated with constitutive activation of certain microRNAs.

### The identified regulatory regions are evolutionarily conserved

**Figure 6:**
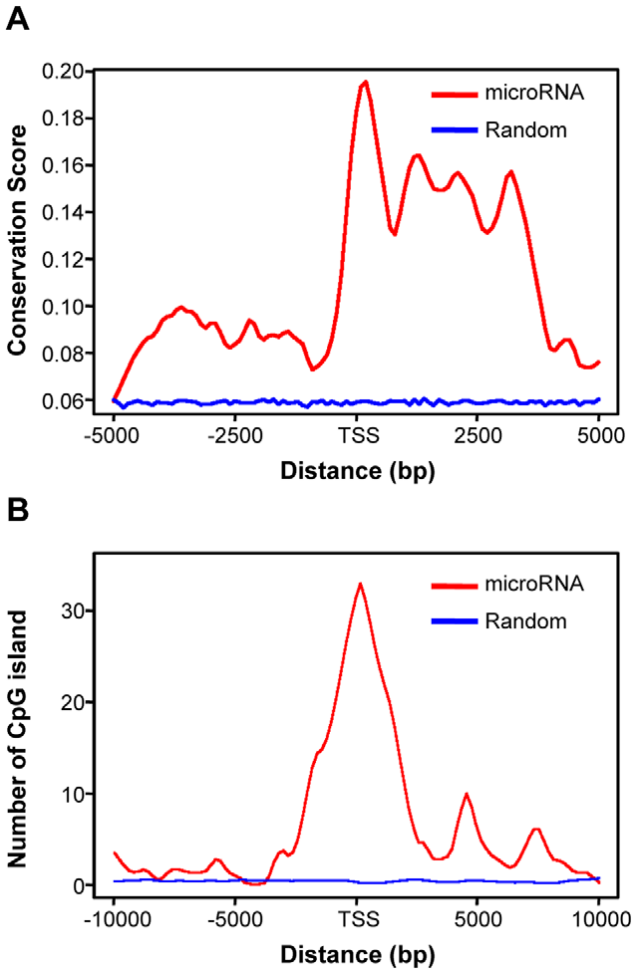
Sequence conservation and CpG islands distribution pattern near the predicted microRNAs TSS and in random intergenic sequences. (A) Sequence conservation around all microRNA TSS in four cell types. (B) CpG islands distribution within 10 kb upstream and downstream of microRNA transcription start sites.

We further examined conservation levels of identified TSS and promoter regions. For each microRNA, the conservation scores (PhastCons scores), were retrieved for five genomic regions (Figure 6A): the 200-bp bin that contained the identified TSS, predicted regulatory regions (Figure 4A), 2,000-bp upstream of the regulatory region, 2,000-bp downstream of the regulatory region, and 2,000-bp of randomly selected intergenic regions. The PhastCons scores are downloaded from UCSC Genome Browser and reflect the overall conservation among seventeen vertebrate species [23]. Importantly, the average conservation score in the TSS region and transcript region are markably higher than the upstream regions.

### The identified microRNA promoters are GC-enriched regions

Approximately 70% of human promoters contain CpG islands [23]. We observed high GC content within or around the predicted regulatory regions, and among the 46 microRNA clusters that contain predicted promoters, 37 (80%) were found to contain or overlap with at least one CpG island; these clusters include 59 out of 72 active microRNAs (Table S2). To examine the distribution of the number of CpG islands at each genomic locus for all the microRNAs, we aligned the identified TSS bin and extended 10,000-bp in both upstream and downstream directions (Figure 6B). We observed clear enrichment of CpG island occupancy around the predicted TSS and regulatory regions.

### Enriched H3K4Me2 signal around the predicted regulatory regions

**Figure 7:**
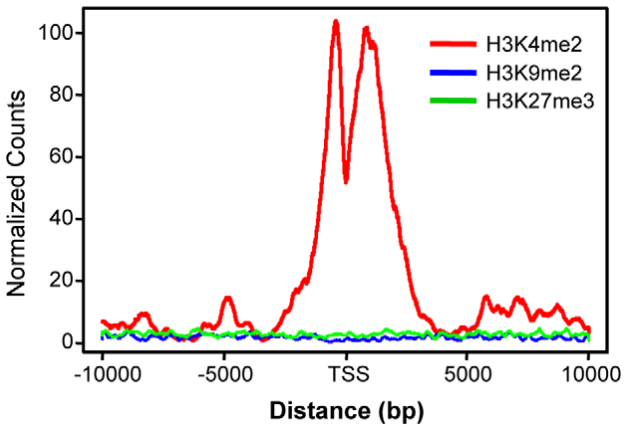
H3K4Me2 binding patterns near the predicted microRNA TSS.

As an independent biological validation, we conducted ChIP-seq experiments on one histone mark, dimethylation of lysine 4 at histone H3 (H3K4Me2). Genome-wide study suggested that this mark localizes around gene promoter and enhancer regions, and forms a bi-peak shape centered at transcription start site [19]. Similar as GC analysis, we aligned the identified TSS bin and extended 10,000-bp in both upstream and downstream directions, and counted the number of H3K4Me2 ChIP-seq fragments on each genomic locus. We observed a bi-peak pattern similar to that reported in [19], [26], [27] (Figure 7).

### Promoter regions for intronic microRNAs

It has been reported that most intronic microRNAs are co-transcribed with their host genes, and therefore share common promoter regions. However, several publications also suggested that some intronic microRNAs could be transcribed independently [18], [28], [29]. To this end, we conducted promoter identification on the intronic microRNAs using our model. Among the 266 intronic microRNAs, our algorithm identified 52 microRNA promoters in at least one of the four conditions (MCF7+/−E2, and MCF7-T+/−E2). Forty nine of the identified promoter regions overlaps with the promoters of their host genes. This is consistent with previous reports. There are, however, three exceptions (hsa-9-1, hsa-miR-935, and hsa-miR-661); their promoter regions locate inside of their host gene. The annotations of the identified promoter and their host genes can be found in Table S3.

## Discussion

High throughput DNA sequencing is rapidly changing the landscape of genomic research [30]. Recent studies using ChIP-seq technology have revealed genome-wide transcription factor binding sites [31], [32], [33], the distribution of histone modifications across the genome [19], and RPol II binding sites and patterns associated with active transcription of coding genes [19], [33]. In this study, we used ChIP-seq-derived RPol II binding data to identify regulatory regions of microRNAs, an important step toward understanding the *cis*-acting element and *trans*-acting factors that control the microRNA expression levels.

We hypothesized that RPol II binding distribution around the TSS is similar for microRNAs and protein coding genes. To test this assumption, we designed a statistical model to characterize RPol II binding patterns using the signals associated with highly expressed coding genes. Briefly, the RPol II ChIP-seq data was used to determine 10 parameters **Φ** that describe 5 Gamma distributions, from which the 5 parameters *S*, *B*, *T*, *K_p_*, and *K_t_* of every expressed coding genes are selected. These 5 parameters determine a Poisson parameter (*λ_ij_*) associated with the distribution of the number of RPol II binding fragments in bin *j* of gene *i*. Rather than being fitted for every expressed gene, these 5 parameters were treated as hidden variables and bounded by five Gamma distributions; this effectively characterized their between gene variations.

To predict the genomic loci of microRNA transcription start sites, we applied the model on the RPol II binding patterns in the upstream region of all annotated microRNAs. We further used this model to investigate the transcription of microRNAs in response to hormone treatment of two breast cancer cell lines, estrogen-dependent breast cancer cells (MCF7) and the anti-estrogen (tamoxifen) resistant subline (MCF7-T). Our model identified TSS for 72 microRNAs in at least one of four conditions (treatment of MCF7 or MCF7-T with either vehicle or 17β-estradiol). Our results suggest that microRNA predisposition can contribute to the development of antiestrogen resistance in hormone-dependent breast cancer cells. It should be noted that while comparing the predictions between two conditions, we did not take the RPol II binding intensity into account; only two states, “active” and “inactive” promoters, were considered. This is to avoid the potential bias caused by the conditional differences between samples, such as sequencing depth, library preparation errors, and so on. It is possible that for certain active promoters, RPol II binding intensity changes but the signal in both conditions are higher comparing to the background (active in both conditions). Our model cannot distinguish such differences. In addition, RPol II enrichment at the promoter region does not guarantee the expression of downstream gene; many mechanisms can contribute to such deviation, such as RPol II stalling, RNA binding protein-induced post-transcriptional regulation, and so on.

Promoter regions and TSS of non-coding RNAs have recently been identified using strategies based on three types of information: 1) sequence composition upstream of the microRNA, such as GC content, level of conservation, transcription factor binding sites and expressed sequence tags [15], [16], [17], [24], [34]; 2) the distribution of epigenetic marks that encode regions of transcriptional initiation [20], [29], or 3) ChIP-chip-derived RPol II binding data using custom tiling arrays designed to target ∼50kb upstream the microRNA genes [18]. Our approach differs from those studies in several ways. First, we did not use sequence composition as the model base for promoter prediction; instead, that type of information is used, in part, for model evaluation. We found that ∼80% of the identified promoter regions overlap with at least one CpG island. In addition, the regions we identified tend to be more evolutionarily conserved. In contrast to sequence information, RPol II binding patterns provide important temporal and spatial measurements regarding the initiation of transcription, important for understanding the mechanism of microRNA transcriptional regulation. Second, our strategy differs from previous efforts using H3K4Me3 marks for successfully identifying microRNA promoter regions [20]. H3K4Me3 highly localizes to promoters [19] and therefore serves as an excellent transcriptional initiation mark. Therefore, we applied our model to one of the datasets containing both H3K4Me3 and RPol II binding data ([19]; from a published study measuring the binding patterns of 20 histone modification markers in human CD4+ T-cells). A detailed comparison between the two strategies revealed several interesting features (Appendix S1), but perhaps most important was that H3K4Me3 maintains a permissive chromatin state that allows for transcription factor binding. However, the permissive chromatin state appears to be necessary, but not sufficient, for transcriptional initiation, as only 23% H3K4Me3-predicted microRNA promoters are recovered by our RPol II strategy (Figure S2). This observation, however, can in part be caused by the differences of experimental conditions, such as sequencing depth. Third, our approach differs from a recent study attempting to identify TSS-containing regions in pri-microRNAs using RPol II ChIP-chip data from a tiling array platform targeting microRNA upstream regions of up to 50KB. Instead of only examining the microRNA upstream RPol II signals, we first trained our model using the RPol II binding patterns around the TSS of protein coding genes, providing a statistical framework for evaluating the sensitivity and specificity of the model prediction (Figure 3). In addition, this framework allows for self-correcting of variable RPol II binding signals from different experiments, due to parameter identification for individual samples, making it possible to compare microRNA promoter signals under different biological conditions.

Despites these advantages, RPol II binding patterns around the TSS can only be used to identify regulatory regions of intergenic microRNAs, which account for approximately half of all microRNAs. Current evidence is lacking as to whether intronic microRNAs use their own TSS and promoter sequences or share the same regulatory components with the host gene. Our results suggest that most of the intronic microRNAs share promoter regions with their host genes, with a few exceptions. Similarly, our TSS search focus on 10kb upstream of microRNA annotation. Recent studies suggest that some microRNA promoters are far away from their mature product on the genome; they will not be predicted by the current strategy. Technically, increasing the searching scope is possible; however, the prediction accuracy will be decreased due to the interference with the RPol II signals of surrounding genes. It should also be noted that the model presented here only focuses on the transcriptional regulation in the microRNA biogenesis process; the microRNA expression can also be affected by other steps, including Drosha-involved nuclear processing [35], [36], nuclear export [35], [37], and Dicer-involved cytomastic processing [35], [36], [37]. In addition, the computational model proposed here cannot be used to identify regulatory regions of the small percentage of microRNAs transcribed by RNA polymerase III [38].

As shown in Eq. 2, the current model did not incorporate the potential correlation among 5 parameters that characterize genome-wide RPol II binding patterns around active promoters. Neglecting such correlations will potentially affect the likelihood estimation, and therefore result in less than optimal promoter prediction. However, ROC curve on our current model suggested that the AUC has reached ∼0.9 in predicting promoter regions of highly expressed genes (Figure 3). Hence, additional improvement with better model won't be significantly beneficial. In order to model the correlations among S, B, and T, at least two more random effects need to be introduced into the model to characterize their shared variations. This additional level of hierarchical model will lead to one more layer of integration in the E-step. The numerical integration scheme will be very different, and computational expense will be much higher. Its complexity will exceed the current scope of this paper, and it is a challenging research question.

Our model differs from regular “peak finder” algorithms that are often used to identify binding sites of transcription factors derived from ChIP-seq experiments. An underlying assumption of regular peak finder algorithms is that DNA-binding proteins, such as transcription factors, contain sequence-specific DNA binding domains that target a cluster of *cis*-acting DNA elements sharing certain sequence features. While such algorithms can identify DNA binding sites for highly specific transcription factors, they are not appropriate for identifying binding sites for the general transcriptional machinery, such as RPol II, which typically does not display high sequence specificity. In addition, as RPol II activity likely extends beyond the promoter/transcription start site of active genes, algorithms for assessing long-range RPol II binding are needed. Our data demonstrated that RPol II binding pattern around the gene transcription start site follows distinct patterns (Figure 2A), and our model is designed to jointly describe the number of RPol II binding fragments surrounding the TSS, including both promoter and transcript regions; this allows for a more accurate description of RPol II binding pattern features. Finally, the model framework described here can also be used to study the activities of other RPol II-related transcriptional events, such as tissue/condition-specific alternative promoter usage [39], bi-directional promoters [40], and regulatory regions of other RPol II-transcribed non-coding RNA in normal and disease states.

## Methods

### ChIP-seq protocol (for both RPol II and H3K4Me2)

Chromatin immunoprecipitation (ChIP) for PoI II (Santa Cruz, sc-899X; 10mg) and H3K4me2 (Upstate, 07-030, 10mg) was performed as previously described [41]. ChIP libraries for sequencing were prepared following standard protocols from Illumina (San Diego, CA) as described in [42]. ChIP-Seq libraries were sequenced using the Illumina Genome Analyzer II (GA II) as per manufacturer's instructions. Sequencing was performed up to 36 cycles for mapping to the human genome reference sequence. Image analysis and base calling were performed with the standard Illumina pipeline, and with automated matrix and phasing calculations on the PhiX control that was run in the eighth lane of each flowcell. Eland_extended algorithm was used to map the sequences to human genome (hg18). This algorithm is fully sensitive to 2 mismatches in first 32 bases and allows up to 6 mismatches in whole read length. Only the sequences that uniquely mapped are reported in export or sorted files.

### Modeling promoter features using coding genes

ChIP-seq experiment revealed that RPol II followed distinct binding patterns around transcription start site of coding genes (Figure 2). In order to model the genome-wide RPol II binding pattern around TSS of coding genes in a statistical framework, we first divided the genomic regions neighboring TSS into 200-bp bins. The bins were classified into three categories, a TSS bin, where the annotated TSS locates in the center of the bin, promoter bins, which locate upstream of the TSS bin, and transcript bins, which locates downstream of the TSS bin. Intuitively, the number of RPol II fragments detected in each bin should follow a Poisson distribution:
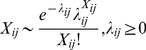
(1)where *X_ij_* denotes the number of detected fragments in the *j*-th bin of the *i*-th gene, and *λ_ij_* is the expected RPol II quantity for the same bin. We assumed that the expected RPol II quantity *λ_ij_* is determined by the expression levels of the *i*-th gene, and the relative location of the *j*-th bin from the transcription start site.
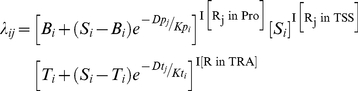
(2)where *S_i_* stands for the expected number of RPol II fragments in the bin that contains the TSS of the *i*-th gene; while *B_i_* and *T_i_* denote the expected RPol II fragments in the bins that locate distantly upstream or downstream of the TSS, which represents the expected signals for the intergenic background and stead transcript regions, respectively (Figure 2B). *D_j_* stands for the distance between the *j*-th bin and the TSS, represented through the number of bins away from the TSS bin. *Kp_i_* and *Kt_i_* denote the decay rate of RPol II signal in the upstream and downstream of TSS of the *i*-th gene. The five parameters, *S_i_*, *B_i_*, *T_i_*, *Kp_i_*, and *Kt_i_*, are all gene specific and are assumed to follow respective *Gamma* distributions genome-wide. The probability of observing the experimentally-determined RPol II binding patterns around the TSS of coding genes can be described as 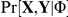, where **X** denotes the number of RPol II fragments observed in each bin; **Y** is missing data that represent five gene specific parameters, *S_i_*, *B_i_*, *T_i_*, *Kp_i_*, and *Kt_i_*; and **Φ** denotes the ten parameters for the *Gamma* distributions of the five missing values. The parameter vector **Φ** was estimated from number of RPol II fragments in each bin around the TSS of the coding genes. See Appendix S1 for details on numerical calculations.

### Identification microRNA regulatory regions

We identified TSS of pri-miRNAs and its regulatory region using the ten parameters **Φ** estimated from RPol II binding patterns surrounding the TSS of coding genes. For each annotated intergenic pre-miRNA in miRBase database, we retrieved the RPol II binding data from 15,000-bp upstream and 5,000-bp downstream of its start genomic locus, allowing for searching for TSS within 10K upstream of the annotated pre-miRNA. As described above, the genomic regions will be divided into a series of 200-bp bins. For each bin, we evaluated the likelihood of containing a TSS by calculating a score that describes the differences between the probability of containing a TSS or not (background); the background model only incorporates hidden value (B) since the gene is assumed not to be expressed
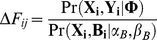
(3)where **Φ** is the estimated parameter vector identified from the RPol II binding data for the coding genes; **X_i_** represents number of RPol II binding fragments in the 50 upstream and downstream bins that surrounding the *j*-th bin (the bin being evaluated). α_B_ and β_B_ represents the two parameters that describing the *Gamma* distribution of genome-wide background signals. See Appendix S1 for detail procedures.

### Data and model availability

All the data are made available in the NCBI Gene Expression Omnibus (GEO) database with accession number GSE21068 for the ChIP-seq data for RPol II and H3K4me2, and GSE5840 for the microarray data for MCF7 and MCF7-T with and without E2 treatment. In addition, both the R-code for the promoter identification and ChIP-seq data are available in the project website: http://compbio.iupui.edu/liu/miRpromoter.

## Supporting Information

Appendix S1 Supplementary methods and results.
(0.14 MB DOC)

Figure S1 The saturation analysis on (A) E2-treated MCF7cells, (B) vehicle MCF7-T cells, (C) E2-treated MCF7-T cells, and (D) CD4^+^ T-cells. Because the gene expression measurements were achieved using different microarray platforms, the expression level for MCF7 and T-cell were sub-classified on different scales.
(0.20 MB TIF)

Figure S2 Congruity between promoter predictions based upon RNA polymerase II and H3K4Me3.
(0.13 MB TIF)

Table S1 The optimal estimations for the 10 parameters in four conditions.
(0.02 MB XLS)

Table S2 The predicted transcription start sites and promoter regions of 72 microRNAs, and their association with CpG islands.
(0.04 MB XLS)

Table S3 Annotations of predicted promoters of intronic microRNAs.
(0.05 MB XLS)
