# Supplementary material for: RNA Polymerase II Binding Patterns Reveal Genomic Regions Involved in MicroRNA Gene Regulation
Source: PLoS One. 2010 Nov 2;5(11):e13798. doi: 10.1371/journal.pone.0013798 (PMC2970572; doi:10.1371/journal.pone.0013798)
Supplement: Appendix S1 — Supplementary methods and results. (0.14 MB DOC) [file pone.0013798.s001.doc]

**Supplementary Methods**

**Rationale**

Using the RNA polymerase II (RPol II) ChIP-seq data, we develop a computational approach to indentify the microRNA promoter region and transcription start site. We observed enrichment of RPol II binding around the transcription start site of coding genes whose expression levels are detectable (Figure 2A). Based upon the assumption that the distribution of RPol II binding patterns around the transcription start site (TSS) of microRNA and protein coding genes are similar, we first indentify the RPol II fragment distribution pattern around TSS of expressed gene, then use identified pattern searching for the promoters and transcription start site upstream of mature microRNA.

**RPol II binding pattern around the TSS of expressed gene**

Centric at transcription start site of coding genes, we divide genomic regions into 200-bp windows (Figure 2B); the window contains transcription start site ranges from -100bp to +100bp. We assume that the number of RPol II fragments falling into each bin follows Poisson distribution.

$X_{ij}\sim\frac{e^{-\lambda_{ij}}\lambda_{ij}^{X_{ij}}}{X_{ij}!},\lambda_{ij}\geq0$ (S1)

where *i* and *j* correspond to gene and bin number, respectively; *X_ij_* denotes the number of detected fragments for *j-th* bin in *i-th* gene; and *_ij_* represents the expected number of RPol II fragments in the same bin. Intuitively, this number is dependent on several factors including the location of the window related to the transcription start site, and the expression levels of the gene. For instance, for a regular gene, we observed highest signal intensity (number of RPol II reads) in the bin where transcription start site appears; such number gradually decreases with the distance to TSS increase, in both upstream and downstream directions. We use 5 parameters to define this pattern (Figure 2B), S – the number of RPol II binding fragments in the central interval (the window where TSS locates); T – the number of RPol II binding fragments in the steady transcript region; B – the number of RPol II binding fragments in the steady background region; and Kp and Kt – decay factors of the number of RPol II binding fragments in the promoter and transcription regions, respectively. These five parameters each follows a Gamma distribution in the genome wide among all the expressed genes:

(S2)

Here, we assume that the RPol II binding patterns around the TSS of expressed genes were determined by 10 Gamma parameters $\Phi=\left[\alpha_{B},\beta_{B},\alpha_{S},\beta_{S},\alpha_{T},\beta_{T},\alpha_{K_{p}},\beta_{K_{p}},\alpha_{K_{t}},\beta_{K_{t}}\right]$; the two parameter *Gamma* distribution is a very flexible function that can describe a wide range of distribution shapes.

In the genomic regions upstream of TSS (promoter), we assume that expected number of RPol II fragments in each bin follows the exponential decreases, with their distances to the TSS increase (Figure 2B):

$\lambda_{P_{ij}}=B_{i}+\left(S_{i}-B_{i}\right)e^{\frac{-Dp_{j}}{Kp_{i}}}$ (S3)

where, *Dp* is the distance from the TSS.

Similarly, in the regions downstream of TSS (transcript region), the expected number of RPol II fragments in each bin follows another exponential decrease, with its distance from TSS increases:

$\lambda_{T_{ij}}=T_{i}+\left(S_{i}-T_{i}\right)e^{\frac{-Dt_{j}}{Kt_{i}}}$ (S4)

where, *Dt* is the distance from the TSS.

The statistical framework is established as a mixture model where *X* denotes incomplete data consisting of the observed number of RPol II fragment, $Y=\left\{B,T,S,Kp,Kt\right\}$ denotes the missing data. Together, *X* and *Y* form the complete data. The joint [probability density function](http://en.wikipedia.org/wiki/Probability_density_function) of the complete data with parameters given by the vector $\Phi=\left[\alpha_{B},\beta_{B},\alpha_{S},\beta_{S},\alpha_{T},\beta_{T},\alpha_{K_{p}},\beta_{K_{p}},\alpha_{K_{t}},\beta_{K_{t}}\right]$ is modeled by the following equation:

$\begin{matrix} \Pr\left(X,Y|\Phi\right) \\ =\Pr\left(X,B,T,S,Kp,Kt|\alpha_{B},\beta_{B},\alpha_{T},\beta_{T},\alpha_{S},\beta_{S},\alpha_{Kp},\beta_{Kp},\alpha_{Kt},\beta_{Kt}\right) \\ =\Pr\left(X|B,T,S,Kp,Kt\right)\Pr\left(B,T,S,Kp,Kt|\alpha_{B},\beta_{B},\alpha_{T},\beta_{T},\alpha_{S},\beta_{S},\alpha_{Kp},\beta_{Kp},\alpha_{Kt},\beta_{Kt}\right) \\ =\prod_{i=1}^{N_{GENE}} \left[\begin{matrix} \prod_{j=1}^{50} \frac{e^{-\lambda_{P_{ij}}}\lambda_{P_{ij}}^{X_{P_{ij}}}}{X_{P_{ij}}!}\bullet\prod_{j=1}^{50} \frac{e^{-\lambda_{T_{ij}}}\lambda_{T_{ij}}^{X_{T_{ij}}}}{X_{T_{ij}}!}\bullet\frac{e^{-\lambda_{TSS_{i}}}\lambda_{TSS_{i}}^{X_{TSS_{i}}}}{X_{TSS_{i}}!} \\ \bullet\frac{Kp_{i}^{a_{Kp}-1}e^{-Kp_{i}/\beta_{Kp}}}{\Gamma(\alpha_{Kp})\beta_{Kp}^{\alpha_{Kp}}}\bullet\frac{Kt_{i}^{a_{Kt}-1}e^{-Kt_{i}/\beta_{Kt}}}{\Gamma(\alpha_{Kt})\beta_{Kt}^{\alpha_{Kt}}}\bullet\frac{B_{i}^{a_{B}-1}e^{-B_{i}/\beta_{B}}}{\Gamma(\alpha_{B})\beta_{B}^{\alpha_{B}}}\bullet\frac{T_{i}^{\alpha_{T}-1}e^{-T_{i}/\beta_{T}}}{\Gamma(\alpha_{T})\beta_{T}^{\alpha_{T}}}\bullet\frac{S_{i}^{\alpha_{S}-1}e^{-S_{i}/\beta_{S}}}{\Gamma(\alpha_{S})\beta_{S}^{\alpha_{S}}} \end{matrix}\right] \end{matrix}$ (S5)

This function can also be considered as the [likelihood](http://en.wikipedia.org/wiki/Likelihood) of the complete data, that is, it can be thought of as a function of . We did not use Expectation-Maximization (EM) algorithm since there is no close form for the E-step. Statistically, the missing value *Y* can be integrated. The integral function is:

$\begin{matrix} \int\Pr(X,Y|\Phi)dY \\ =\prod_{i=1}^{N_{GENE}} \int_{0}^{+\infty} \left\{\begin{matrix} \left(\frac{e^{-\lambda_{TSS_{i}}}\lambda_{TSS_{i}}^{X_{TSS_{i}}}}{X_{TSS_{i}}!}\bullet\frac{S_{i}^{a_{S}-1}e^{-S_{i}/\beta_{S}}}{\Gamma(\alpha_{S})\beta_{S}^{\alpha_{S}}}\right)\bullet\int_{0}^{+\infty} \int_{0}^{+\infty} \frac{Kp_{i}^{a_{Kp}-1}e^{-Kp_{i}/\beta_{Kp}}}{\Gamma(\alpha_{Kp})\beta_{Kp}^{\alpha_{Kp}}}\bullet\frac{B_{i}^{a_{B}-1}e^{-B_{i}/\beta_{B}}}{\Gamma(\alpha_{B})\beta_{B}^{\alpha_{B}}}\bullet\prod_{j=1}^{50} \frac{e^{-\lambda_{P_{ij}}}\lambda_{P_{ij}}^{X_{P_{ij}}}}{X_{P_{ij}}!}dK_{Pi}dB_{i}\bullet\\ \int_{0}^{+\infty} \int_{0}^{+\infty} \frac{Kt_{i}^{a_{Kt}-1}e^{-Kt_{i}/\beta_{Kt}}}{\Gamma(\alpha_{Kt})\beta_{Kt}^{\alpha_{Kt}}}\bullet\frac{T_{i}^{\alpha_{T}-1}e^{-T_{i}/\beta_{T}}}{\Gamma(\alpha_{T})\beta_{T}^{\alpha_{T}}}\bullet\prod_{j=1}^{50} \frac{e^{-\lambda_{T_{ij}}}\lambda_{T_{ij}}^{X_{T_{ij}}}}{X_{T_{ij}}!}dK_{Ti}dE_{i} \end{matrix}\right\}dS_{i} \\ =\prod_{i=1}^{N_{GENE}} \int_{0}^{+\infty} \left\{\begin{matrix} \left(\frac{e^{-\lambda_{TSS_{i}}}\lambda_{TSS_{i}}^{X_{TSS_{i}}}}{X_{TSS_{i}}!}\bullet\frac{S_{i}^{a_{S}-1}e^{-S_{i}/\beta_{S}}}{\Gamma(\alpha_{S})\beta_{S}^{\alpha_{S}}}\right)\bullet\int_{0}^{+\infty} \frac{B_{i}^{a_{B}-1}e^{-B_{i}/\beta_{B}}}{\Gamma(\alpha_{B})\beta_{B}^{\alpha_{B}}}\bullet\int_{0}^{+\infty} \frac{Kp_{i}^{a_{Kp}-1}e^{-Kp_{i}/\beta_{Kp}}}{\Gamma(\alpha_{Kp})\beta_{Kp}^{\alpha_{Kp}}}\bullet\prod_{j=1}^{50} \frac{e^{-\lambda_{P_{ij}}}\lambda_{P_{ij}}^{X_{P_{ij}}}}{X_{P_{ij}}!}dK_{P_{i}}dB_{i}\bullet\\ \int_{0}^{+\infty} \frac{T_{i}^{\alpha_{T}-1}e^{-T_{i}/\beta_{T}}}{\Gamma(\alpha_{T})\beta_{T}^{\alpha_{T}}}\bullet\int_{0}^{+\infty} \frac{Kt_{i}^{a_{Kt}-1}e^{-Kt_{i}/\beta_{Kt}}}{\Gamma(\alpha_{Kt})\beta_{Kt}^{\alpha_{Kt}}}\bullet\prod_{j=1}^{50} \frac{e^{-\lambda_{T_{ij}}}\lambda_{T_{ij}}^{X_{T_{ij}}}}{X_{T_{ij}}!}dK_{Ti}dE_{i} \end{matrix}\right\}dS_{i} \end{matrix}$ (S6)

**Integration process**

The integral form in Equation S6 is complicated and computationally challenging. Considering the computation cost and precision, we use a 5-order Laguerre integration function (Stroud and Secrest, 1967) to approximate the integration form. The Laguerre function is described as:

$\int_{0}^{\infty} e^{-x}f\left(x\right)dx\approx\sum_{i=1}^{n} w_{i}f\left(x_{i}\right)$ (S7)

where, x_i_ is Zero of Laguerre Polynomials, w_i_ is weight factor of Laguerre. In this function, we use n as 5 (Stro*ud et* al, 1967). From the Laguerre integration table, the values of {*x_1_, x_2_, x_3_, x_4_, x_5_*} are {0.26, 1.41, 3.60, 7.09, 12.64}, and the values of {*w1, w2, w3, w4, w5*} are {0.52, 0.40, 0.076, 0.0036, 0.000023}.

The integration of different integral items was processed in sequential. The final form can be derived:

$\begin{matrix} \int\Pr(X,Y|\Phi)dY \\ =\prod_{i=1}^{N_{GENE}} \sum_{s=1}^{n} w_{s}\bullet\left(\begin{matrix} \frac{e^{-y_{s}\beta_{S}}\left(y_{s}\beta_{S}\right)^{X_{TSS_{i}}}\left(y_{s}\beta_{S}\right)^{a_{S}-1}\beta_{S}}{X_{TSS_{i}}!\Gamma(\alpha_{S})\beta_{S}^{\alpha_{S}}}\bullet\frac{\beta_{Kp}\beta_{B}}{\Gamma(\alpha_{B})\beta_{B}^{\alpha_{B}}\Gamma(\alpha_{Kp})\beta_{Kp}^{\alpha_{Kp}}\prod_{j=1}^{50} X_{P_{ij}}!}\bullet\frac{\beta_{Kt}\beta_{E}}{\Gamma(\alpha_{E})\beta_{E}^{\alpha_{E}}\Gamma(\alpha_{Kt})\beta_{Kt}^{\alpha_{Kt}}\prod_{j=1}^{50} X_{T_{ij}}!} \\ \bullet\sum_{b=1}^{n} w_{b}\bullet\left(\sum_{p=1}^{n} w_{p}\left(\left(y_{b}\beta_{B}\right)^{a_{B}-1}\bullet\left(y_{p}\beta_{Kp}\right)^{a_{Kp}-1}\bullet\prod_{j=1}^{50} e^{-y_{b}\beta_{B}-\left(y_{s}\beta_{S}-y_{b}\beta_{B}\right)e^{\frac{-D_{Pj}}{y_{p}\beta_{Kp}}}}\left(y_{b}\beta_{B}+\left(y_{s}\beta_{S}-y_{b}\beta_{B}\right)e^{\frac{-D_{Pj}}{y_{p}\beta_{Kp}}}\right)^{X_{P_{ij}}}\right)\right) \\ \bullet\sum_{e=1}^{n} w_{e}\bullet\left(\sum_{t=1}^{n} w_{t}\left(\left(y_{e}\beta_{E}\right)^{a_{E}-1}\bullet\left(y_{t}\beta_{Kt}\right)^{a_{Kt}-1}\bullet\prod_{j=1}^{50} e^{-y_{e}\beta_{E}-\left(y_{s}\beta_{S}-y_{e}\beta_{E}\right)e^{\frac{-D_{Tj}}{y_{t}\beta_{Kt}}}}\left(y_{e}\beta_{E}+\left(y_{s}\beta_{S}-y_{e}\beta_{E}\right)e^{\frac{-D_{Tj}}{y_{t}\beta_{Kt}}}\right)^{X_{T_{ij}}}\right)\right) \end{matrix}\right) \end{matrix}$ (S8)

After the integration of missing values {*B, T, S, Kp, Kt*}, the unknown values in equation S8 are $\Phi=\left[\alpha_{B},\beta_{B},\alpha_{S},\beta_{S},\alpha_{T},\beta_{T},\alpha_{K_{p}},\beta_{K_{p}},\alpha_{K_{t}},\beta_{K_{t}}\right]$.

**Optimization procedure**

The parameters in the *Gamma* distribution $\Phi=\left[\alpha_{B},\beta_{B},\alpha_{S},\beta_{S},\alpha_{T},\beta_{T},\alpha_{K_{p}},\beta_{K_{p}},\alpha_{K_{t}},\beta_{K_{t}}\right]$ are estimated by maximizing the likelihood function described in Equation S8. Since searching for the optimal solutions for the *Gamma* parameters can be trapped into local optimum which causes either slow convergence or fails to converge to the global optimal solution. Here we utilized particle swarm optimization (PSO) (Parsopoulos and Vrahatis, 2002), an artificial intelligence approach that mimics a behavior of swarm-forming agents, which provides a good balance between global optimum searching and computation efficiency. The PSO optimization procedures are conducted with the following four steps.

Step 1: one thousand particles (potential solution) were initially randomly distributed in a 10-dimensional parameter spaces $(\alpha_{B},\beta_{B},\alpha_{S},\beta_{S},\alpha_{T},\beta_{T},\alpha_{K_{p}},\beta_{K_{p}},\alpha_{K_{t}},\beta_{K_{t}})$.

Step 2: the likelihood of each of the 1000 particles are calculated following Equation S8.

Step 3: the velocity vector of the particle, serving as the guidance to search for the optimal solution, was calculated using Equation S9.

$\mathbf{V}_{k}^{'}=c_{0}\mathbf{V}_{k}+c_{1}(\mathbf{P}_{global}-\mathbf{P}_{k})+c_{2}(\mathbf{P}_{k-local}-\mathbf{P}{}_{k})$ (S9)

where **P***_global_* is the global optimal solution achieved so far; **P***_k-local_* is local optimal solution achieved by particle *k*; and *C_0_*, *C_1_*, *C_2_* are weight factors that are adjustable to control the searching speed.

Step 4: in the solution space, all the particles are re-positioned based on their current positions and movement velocities calculated in Equation S10.

$\mathbf{P}_{k}^{'}=\mathbf{P}_{k}+\mathbf{V}_{k}^{'}$ (S10)

Steps 1 through 4 will be iterated until further particle movement cannot result in higher likelihood defined in Equation S8.

In the practical calculation, the PSO optimization procedure is efficient; it converges in 25 to 30 cycles.

**Differential RPol II binding between the active promoter region and other regions**

In the Equation S5, the likelihood of detected RPol II binding tags in the promoter is calculated by the assumption that the RPol II binding patterns are different in the random intergentic regions (background) and genetic regions approximating to the TSS of expressed genes (promoter region and transcription region combined). Therefore, we assume that the number of RPol II binding fragments in each bin in the random intergentic regions follows the same *Poisson* distribution with the background region.

$\Pr\left(X,B|\alpha_{B},\beta_{B}\right)=\prod_{i=1}^{N_{GENE}} \left[\frac{B_{i}^{a_{3}-1}e^{-B_{i}/\beta_{3}}}{\Gamma(\alpha_{3})\beta_{3}^{\alpha_{3}}}\bullet\prod_{j=1}^{101} \frac{e^{-B_{i}}B_{i}^{X_{ij}}}{X_{ij}!}\right]$ (S11)

Similar as function S5, when estimating the likelihood that the tested genomic regions fell into background regions, we use Laguerre integration function to integrate the missing value B. The integral function can be approximated:

$\begin{matrix} \int_{0}^{+\infty} \prod_{i=1}^{N_{GENE}} \left[\frac{B_{i}^{a_{B}-1}e^{-B_{i}/\beta_{B}}}{\Gamma(\alpha_{B})\beta_{B}^{\alpha_{B}}}\bullet\prod_{j=1}^{101} \frac{e^{-B_{i}}B_{i}^{X_{ij}}}{X_{ij}!}\right]dB_{i} \\ =\prod_{i=1}^{N_{GENE}} \sum_{b=1}^{n} w_{b}\bullet\left(\frac{1}{\Gamma(\alpha_{B})\beta_{B}^{\alpha_{B}}X_{ij}!}\bullet\left(y_{b}\beta_{B}\right)^{a_{B}-1}\prod_{j=1}^{101} e^{-y_{b}\beta_{B}}\left(y_{b}\beta_{B}\right)^{X_{ij}}\right) \end{matrix}$ (S12)

where all parameters are same with the function S8.

In order to estimate the difference between them, we devised a score ΔF, representing the difference between the score that matches to expressed gene promoter pattern (Eq. S8) and the score following the same *Poisson* distribution with the background regions since the gene is assumed not to be expressed, Eq. S12). ΔF will calculated by the following equation:

$\Delta F=\frac{\Pr\left(X,B,T,S,Kp,Kt|\alpha_{B},\beta_{B},\alpha_{T},\beta_{T},\alpha_{S},\beta_{S},\alpha_{Kp},\beta_{Kp},\alpha_{Kt},\beta_{Kt}\right)}{\Pr\left(X,B|\alpha_{B},\beta_{B}\right)}$ (S13)

For candidate promoter, a larger ΔF means great probability to appear as a promoter.

**microRNA regulatory region identification**

We use the parameters trained based on RPol II pattern around TSS of protein-coding genes to identify the microRNA regulatory region. In this step, we retrieve the pre-miRNA upstream 15000 bp and downstream 5000 bp and separate into 200-bp bins. We search the 10kb upstream of all 419 pre-microRNA for TSS by using 51 sliding windows (1 window containing TSS, and 25 windows upstream and downstream of TSS windows, respectively). The region which has the maximal ΔF score was identified as the microRNA promoter candidate. In order to estimate the length of microRNA promoter, the missing data of each microRNA, B, T, S, Kp, Kt, was calculated by the PSO approach to maximize the likelihood function S6. We define the microRNA promoter region which the RPol II binding quantity descend 90% of the level in the bin that contains TSS (Figure 4A).

**Supplementary results**

**Comparison with H3K4Me3 strategy**

Marson et.al (Mars*on et* al, 2008) reported a strategy to identify the microRNA transcription start site, based upon H3K4Me3 binding patterns. They considered the H3K4Me3 enriched region as TSS of microRNA. Using H3K4Me3 high-resolution ChIP-seq and ChIP-ChIP data in multiple cell types, promoters of 294 human pri-miRNAs (including 441 mature microRNAs) were identified. Specifically in human CD4+ T-cells, 224 promoters (associating with 243 pri-microRNAs) were identified, of which 126 locate in intergenic regions. In order to compare the differences of our approach (using RPol II binding patterns) with the one using H3K4Me3 strategy, we applied our method using the RPol II ChIP-seq data in the same cell line to predict microRNA promoters, through which promoters of 36 intergenic microRNAs were predicted. The congruity between two strategies is shown in Figure S2.

Comparing to Marson et al, RPol II strategy predicted a subset of microRNA promoters by H3K4Me3 approach. There are totally 29 microRNAs predicted by both two strategies, of which promoter regions of 21 microRNAs overlaps. Among the 8 microRNAs that were predicted by both strategies, but with non-overlapping promoter regions, the predictions based upon RPol II strategy are clearly more biologically relevant. Among these 8 predictions, the promoter of six microRNAs, hsa-mir-142, hsa-mir-23a, hsa-mir-24-2, hsa-mir-27a, hsa-mir-658 and hsa-mir-92b were predicted in the downstream of these microRNAs using the method based on H3K4Me3. In addition, hsa-mir-21 was identified the same promoter with a protein coding gene, TMEM49, which was 135k-bp away from mature mir-21, while our model based upon RPol II binding patterns selected regions that are 2k-4k upstream of mature mir-21. The promoter prediction of hsa-mir-21 was consistent with another microRNA promoter approach (Fujita and Iba, 2008), which used the conservation information comparing multiple species.

Our model identified 7 microRNA promoters which were not predicted in CD4+ T-cells but predicted in other cell types by the approach used in Marson et al (Mars*on et* al, 2008). We manually checked the RPol II and H3K4Me3 signals of CD4+ T-cells in the predicted promoter regions of 7 microRNAs. Interestingly, in the predicted promoter of hsa-mir-192 and hsa-mir-194-2 histone H3 was less trimethylated at its lysine 4 residue, but more RNA polymerase II was detected in CD4+ T-cells. The promoters of hsa-mir-374a, hsa-mir-505 and hsa-mir-545 were predicted in the same regions by our approach in CD4+ T-cells and Marson’s approach in other cell types, but we manually found that the promoters of these 3 microRNAs had enriched H3K4Me3 signals in CD4+ T-cells.

**Reference in supplementary information**

Fujita S, Iba H (2008) Putative promoter regions of miRNA genes involved in evolutionarily conserved regulatory systems among vertebrates. *Bioinformatics* 24: 303-308.

Marson A, Levine SS, Cole MF, Frampton GM, Brambrink T, Johnstone S, Guenther MG, Johnston WK, Wernig M, Newman J, Calabrese JM, Dennis LM, Volkert TL, Gupta S, Love J, Hannett N, Sharp PA, Bartel DP, Jaenisch R, Young RA (2008) Connecting microRNA genes to the core transcriptional regulatory circuitry of embryonic stem cells. *Cell* 134: 521-533.

Parsopoulos KE, Vrahatis MN (2002) Recent approaches to global optimization problems through Particle Swarm Optimization. *Natural Computing* 1: 235-306.

Stroud AH, Secrest D (1967) Gaussian Quadrature Formulas. *Mathematics of Computation* 21: 125-126.
