# Supplementary figures and images for: RNA Polymerase II Binding Patterns Reveal Genomic Regions Involved in MicroRNA Gene Regulation
Source: PLoS One. 2010 Nov 2;5(11):e13798. doi: 10.1371/journal.pone.0013798 (PMC2970572; doi:10.1371/journal.pone.0013798)

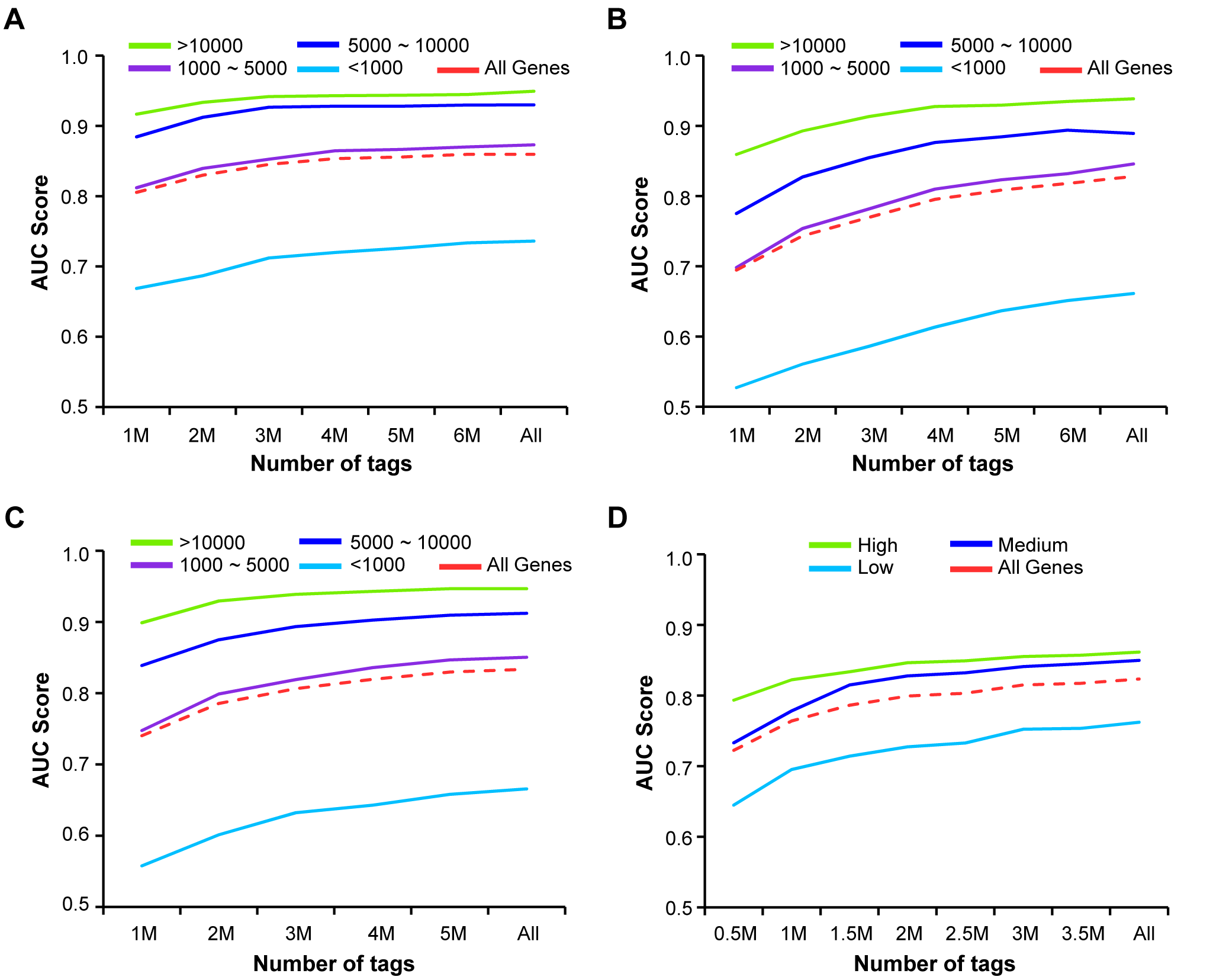

Supplement: Figure S1 — The saturation analysis on (A) E2-treated MCF7cells, (B) vehicle MCF7-T cells, (C) E2-treated MCF7-T cells, and (D) CD4+ T-cells. Because the gene expression measurements were achieved using different microarray platforms, the expression level for MCF7 and T-cell were sub-classified on different scales. (0.20 MB TIF) [file pone.0013798.s002.tif]

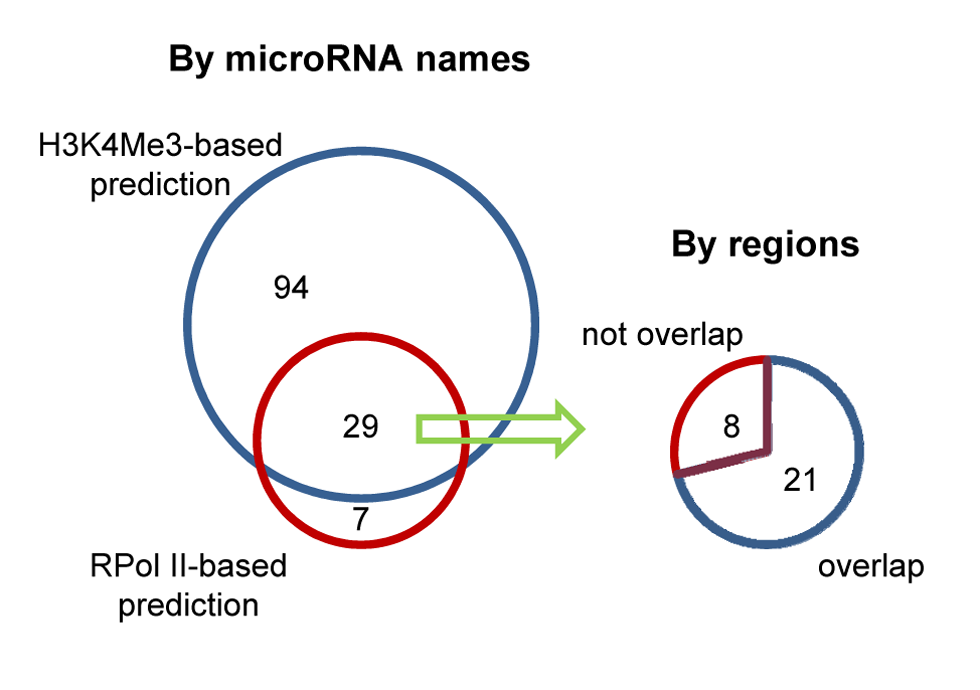

Supplement: Figure S2 — Congruity between promoter predictions based upon RNA polymerase II and H3K4Me3. (0.13 MB TIF) [file pone.0013798.s003.tif]
